# Clinical Differences between SARS-CoV-2 and RSV Infections in Infants: Findings from a Case–Control Study

**DOI:** 10.3390/v16010063

**Published:** 2023-12-30

**Authors:** Victor Daniel Miron, Raluca-Oana Raianu, Claudiu Filimon, Mihai Craiu

**Affiliations:** 1 Carol Davila University of Medicine and Pharmacy, 050474 Bucharest, Romania; 2 National Institute for Mother and Child Health “Alessandrescu-Rusescu”, 020395 Bucharest, Romania

**Keywords:** infant, SARS-CoV-2, COVID-19, RSV, clinical features, respiratory failure, laryngitis

## Abstract

Infants are a unique pediatric group due to their high hospitalization rates and unfavorable outcomes from acute infectious diseases. Understanding the clinical differences and aftereffects of SARS-CoV-2 in comparison to other prevalent viruses in this age group, like RSV, is crucial for effective management. We conducted a retrospective case–control study of infants hospitalized with SARS-CoV-2 or respiratory syncytial virus (RSV) infection in one year, in a tertiary pediatric hospital in Bucharest, Romania. A total of 188 infants were included in the analysis in a 1:1 ratio (94 with SARS-CoV-2 infection and 94 with RSV infection). Infants with COVID-19 were 10.2 times more likely to have fever (*p* < 0.001) and 2.4 times more likely to have diarrhea (*p* = 0.016). Conversely, infants with RSV were 2.5 times more likely to have a cough (*p* < 0.001), 3.0 times more likely to have nasal congestion (*p* < 0.001), and 14.7 times more likely to present with dyspnea (*p* < 0.001). Increased lymphocyte count was more common in infants with RSV (*p* = 0.008), while lymphopenia was more frequent in infants with SARS-CoV-2 (*p* = 0.011). The median length of hospital stay was one day longer in infants with RSV infection (5 days vs. 4 days). Overall, infants with RSV infection had a 27.3-fold increased risk of developing respiratory failure (*p* < 0.001), while infants with COVID-19 had a 5.8-fold increased risk of laryngitis (*p* = 0.003). Our findings suggest that infants with SARS-CoV-2 infection may present with polymorphic symptoms, mostly dominated by fever, whereas infants with RSV often present with respiratory symptoms. Laboratory differentiation between the two infections is challenging; therefore, the use of rapid antigen or molecular diagnostic tests is crucial for accurate diagnosis, epidemiologically appropriate measures, and effective management. Continued surveillance of both viruses in infants, and beyond, and the implementation of specific control measures are needed to mitigate their impact on this vulnerable pediatric group.

## 1. Introduction

The first year of life is an extremely important period in a child’s development. Any external factor, especially acute illnesses, can interfere with and disrupt the developmental process. In addition, an untrained and still evolving immune system puts infants at risk of an unfavorable outcome in the event of an acute infectious disease [1]. Therefore, this group of children requires close monitoring to prevent potential complications.

Based on experience with influenza and respiratory syncytial virus (RSV) infections, where young children are at risk of hospitalization and potentially severe outcomes, the emergence of circulating SARS-CoV-2 was viewed with special concern for this group of children [2].

More than three years after the onset of the COVID-19 pandemic, it has been observed that SARS-CoV-2 infection has had a significant impact on the pediatric population, and although the majority of cases have had favorable outcomes [3], there is still a need for further research; there have also been intensive care unit (ICU) admissions and deaths associated with COVID-19. In an analysis of causes of death among children and young people aged 0 to 19 years in the United States, SARS-CoV-2 infection was included in the top ten, precisely ranking eighth [4].

The true impact of SARS-CoV-2 infection in infants has not been fully quantified and characterized, as data for this age group are clearly lacking. A good way to fully characterize COVID-19 in infants is to compare it with other viral infections with a significant impact on this population, in particular RSV infection. RSV is known to be the leading cause of hospitalization and bronchiolitis in infants [5].

In this context, we aimed to conduct a case–control study describing the clinical course and evolution of COVID-19 in hospitalized infants by conducting a comparative analysis with infants with RSV infection.

## 2. Methods

We conducted a retrospective case–control study among infants hospitalized with SARS-CoV-2 or RSV infection at the National Institute for Mother and Child Health “Alessandrescu-Rusescu” (NIMCH) over a 12-month period from April 2021 to March 2022 (Delta and Omicron SARS-CoV-2 variants).

NIMCH is one of four university children’s hospitals in Bucharest, the main city of Romania. Approximately 10,000 children are admitted to the hospital each year, and more than 60,000 children are seen in the outpatient and/or emergency departments. At the beginning of the COVID-19 pandemic, NIMCH was designated as the hospital to treat non-COVID-19 cases, mainly respiratory and digestive pathology. Since April 2021, an eight-bed ward has been opened in the hospital for the admission of children with SARS-CoV-2 infection.

Cases in this study were specifically identified as infants who were hospitalized during the study period and confirmed to have SARS-CoV-2 infection by reverse transcription-polymerase chain reaction (RT-PCR) testing. We included all cases consecutively, excluding children older than 1 year at the time of hospitalization, those with incomplete electronic data, and those with other co-infections as evidenced by rapid tests, multiplex RT-PCR, or cultures. This approach was taken to ensure that our study focused exclusively on cases infected with SARS-CoV-2 only.

Controls were defined as infants with RSV infection confirmed by rapid antigen or multiplex RT-PCR testing who were hospitalized during the study period. We matched the controls to the cases consecutively by sex and age group. Exclusions were similar to the cases: children over 1 years old at hospitalization, those lacking complete electronic data, and those with other proven co-infections (similar to that described above for the cases).

The case:control ratio was set at 1:1. The equal division of the study participants into these two groups allowed for a balanced comparison, ensuring that each group was adequately represented in the analysis. This parity is essential for a fair and accurate comparison between the two infections, providing insights into their similarities and differences in terms of symptoms, severity, and overall impact on the health of infants.

For each infant identified as a case or control in the study, a complete set of data including demographic and clinical data, laboratory parameter values, and outcomes was collected. In order to facilitate a more detailed and nuanced analysis, the infants in our study were categorized into five distinct age groups. This segmentation was based on their age at the time of their involvement in the study: newborn (0–28 days), 1–3 months, 4–6 months, 7–9 months, and 10–12 months.

A statistical analysis was performed using IBM SPSS Statistics software, version 25 (IBM Corp., Armonk, NY, USA). The level of statistical significance was set at *p* < 0.05. Categorical data were compared using the chi-squar (χ^2^) test, and risk was reported as the odds ratio (OR) along with the 95% confidence interval (95%CI). Continuous variables were analyzed using the Mann–Whitney-U (U) test with z-value (z) and effect size (r) because their distribution was non-Gaussian.

## 3. Results

A total of 188 infants were included in the analysis, equally divided into two groups of 94 each, one with SARS-CoV-2 infection and the other with RSV infection. The demographic characteristics of both groups were comparable and are summarized in Table 1.

An epidemiological context was more commonly identified among infants with COVID-19 (51.1%, *n* = 48) than those with RSV infection (36.2%, *n* = 34, *p* = 0.039, χ^2^ = 61.3, OR = 0.5, 95%CI: 0.3–0.97), particularly in the 1–3 and 4–6 months age groups (see Supplementary Materials).

A total of 22 (11.7%) infants included in the study had at least one chronic disease. Of these infants, 9 had SARS-CoV-2 infection and 13 had RSV infection (*p* = 0.364). Congenital heart diseases were most commonly identified (10 infants, 4 COVID-19, and 6 RSV), followed by genetic diseases (8 infants, 5 COVID-19, and 3 RSV), neurological diseases (8 infants, 3 COVID-19, and 5 RSV), and pulmonary diseases (4 infants, all with RSV infection).

Infants with SARS-CoV-2 infection generally presented to the hospital earlier than those with RSV infection. The median hospital presentation was 1 day for COVID-19 (IQR: 1.0–2.3 days) versus 3 days for RSV infection (IQR: 2.0–4.0 days), *p* < 0.001, z = −6.997, U = 1878.5.

Significant clinical differences were observed between the two infections. Infants with COVID-19 were 10.2 times more likely to have fever (*p* < 0.001, OR = 10.2) and 2.4 times more likely to have diarrhea (*p* = 0.016, OR = 2.4). Conversely, infants with RSV were 2.5 times more likely to have a cough (*p* < 0.001, OR = 0.5), 3.0 times more likely to have nasal congestion (*p* < 0.001, OR = 0.3), and 14.7 times more likely to present with dyspnea (*p* < 0.001, OR = 0.07) (Table 2). In the age group analysis (Supplementary Materials), we found that cough and nasal congestion were significant in infants younger than 6 months with RSV, whereas fever was significantly predominant in infants aged 1–9 months with COVID-19.

In terms of laboratory changes, an increase in monocytes was the most common finding in both groups (83.0% and 80.9%, respectively, *p* = 0.705, Table 3). However, increased lymphocyte counts were more common in infants with RSV infection (*p* = 0.008, OR = 0.5), while decreased lymphocyte counts were more frequent in infants with SARS-CoV-2 infection (*p* = 0.011, OR = 3.1). Additionally, infants with COVID-19 were 2.5 times more likely to have an inflammatory syndrome characterized by elevated C-reactive protein levels compared to those with RSV infection (*p* = 0.007, OR = 2.6). Table 3 summarizes the laboratory changes analyzed between the two groups. By age group, the trend of changes was similar to those highlighted above, and they are presented in the Supplementary Material.

A reduced number of only 31 infants (31/188, 16.5%) required imaging investigations such as chest X-ray (19 infants (20.2%) with COVID-19 and 12 infants (12.8%) with RSV infection, *p* = 0.169). In 54.8% of cases (17/31), these infants showed interstitial pneumonia, and in 41.9% (13/31), they were normal without any pathological changes. Normal appearance was more common in SARS-CoV-2 infection (57.9% vs. 16.7%, *p* < 0.001, OR = 6.9), Table 3.

**Figure 1:**
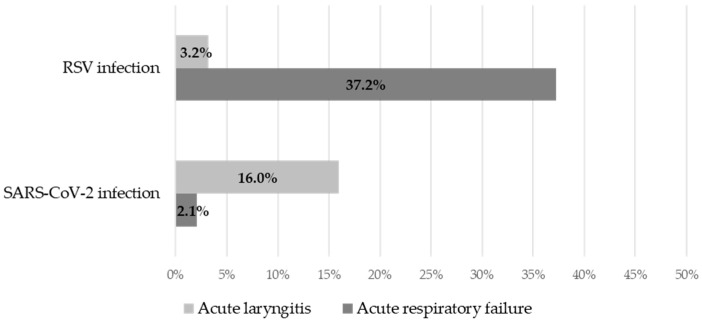
Rates of acute respiratory failure and acute laryngitis by group.

The median length of hospital stay was 1 day longer in infants with RSV infection: 5 days (IQR:3, 7 days) versus 4 days (IQR:3, 7 days) for COVID-19, *p* = 0.079, z = −1.756, U = 3768.0. The presence of acute respiratory failure in infants with RSV significantly increased the median length of hospital stay by 3 days (*p* < 0.001). The outcome was favorable for all cases, with no deaths in either group. Overall, infants with RSV infection had a 27.3-fold increased risk of respiratory failure (2.1%, *n* = 2 vs. 37.2%, *n* = 35, *p* < 0.001, χ^2^ = 36.6, OR = 0.04, 95%CI: 0.008–0.16), whereas infants with COVID-19 had a 5.8-fold increased risk of acute laryngitis (16.0%, *n* = 15 vs. 3.2%, *n* = 3, *p* = 0.003, χ^2^ = 8.8, OR = 5.8, 95%CI: 1.6–20.6) (Figure 1). The incidence of acute dehydration syndrome was relatively similar between the two groups (56.4%, *n* = 53 for SARS-CoV-2 vs. 47.9%, *n* = 45 for RSV, *p* = 0.243). Only five cases were complicated by acute otitis media, one case in an infant with COVID-19 and four cases in children with RSV (*p* = 0.174).

## 4. Discussion

We conducted a case–control analysis to highlight the characteristics of SARS-CoV-2 infection in contrast to RSV infection in hospitalized infants over a period of one year in a pediatric teaching hospital in Bucharest. Respiratory infections are responsible for an increased morbidity and mortality rate among the pediatric population. RSV is one of the most incriminated viruses involved, among young children, especially infants [6]. It has been observed that 45% of hospitalizations due to RSV are reported in children under 6 months of age, which is associated with a risk of unfavorable outcomes with respiratory failure [7]. Studies have also shown that the majority of infants with RSV requiring hospitalization were previously healthy and born at term [8], while premature children or children with certain underlying medical conditions are at higher risk of ICU admission [9]. In contrast, SARS-CoV-2 infection in children resulted in mild-to-moderate disease. Hospitalizations have been reported mainly in young children and infants, mainly for medical follow-up, as the natural course of the disease is benign [10]. However, ICU admissions were also reported among infants with COVID-19, but the admission rate was less than 2% [11].

A European (including data from our country) surveillance study of epidemiology aspects during the SARS-CoV-2 pandemic [12] documented a decrease in RSV circulation to a low rate of positivity, 1% of specimens, from more than 21,000 primary care sentinel surveillance centers. Only France and Switzerland documented clear waves of RSV activity, and France, Germany, and Slovenia recorded an early start to their seasonal 2021–2022 circulation compared with the average starting week in pre-COVID-19 pandemic seasons [12]. This important change in circulation paradigm was also documented in other parts of the world, where RSV circulates during the winter months, such as in Australia and New Zealand [13] and South Africa [14].

One particular aspect of RSV circulation in our country, during the study period, was the lack of prophylactic measures for severe RSV infections in vulnerable populations. Former premature children are at risk of developing respiratory failure during an RSV episode, as described by pre-pandemic papers from our country [15]. From 2022, palivizumab (monoclonal anti-RSV antibodies) became available again, after more than a decade of unattainability, for these children (premature infants with a shorter gestational age than 35 weeks, children younger than 2 years of age with bronchopulmonary dysplasia or with severe congenital heart disease, and infants with cystic fibrosis, neuromuscular disorders or immune-deficit syndromes [16]). Protection for severe RSV infection, in Romanian children, will be increased in the near future through the use of nirsevimab, after European Medicines Agency (EMA) approval in September 2022 [17].

In our analysis, we included cases of the COVID-19 variants Delta and Omicron, when both in Romania [10], and other countries [18], the number of cases of SARS-CoV-2 infection in children was increasing. Infants with RSV infection were selected from the same period by matching cases according to sex and age for robust conclusions regarding our data.

The specific prevalence of the SARS-CoV-2 virus within our country can likely be attributed to multiple contributing factors. One significant aspect is the relatively low rate of vaccination among eligible individuals, with only 42.2% of the general population and 50.7% of those aged 18 and above being vaccinated [19]. These figures are notably lower compared to the European Union/European Economic Area (EU/EEA) average, which stands at 73%. This underwhelming vaccination uptake is indicative of deeper societal issues, such as a widespread lack of trust in governmental institutions, the influence of misinformation campaigns, inadequate infrastructure in rural areas, and a deficiency in effective vaccine education and outreach efforts [20,21,22]. These factors have collectively played a role in shaping the country’s response to the pandemic and the virus’s circulation.

Infants with COVID-19 most commonly presented to hospital with fever (81.9%), with or without respiratory symptoms such as cough (68.1%) or nasal congestion (47.9%). One third of cases had gastrointestinal symptoms, with diarrhea being the most common. In another Romanian study of 613 infants hospitalized with SARS-CoV-2 infection, fever was also the main symptom (96.4%), followed by cough (64.8%), and diarrhea was present in 37.5% of children [10]. In an analysis from Poland of 300 hospitalized cases of COVID-19 in infants, similar data were reported with a frequency of 77% fever, 40% cough, and 24% diarrhea [23].

In contrast, the infants with RSV in the study had a clinical picture dominated by respiratory symptoms, with cough present in all cases, followed by nasal congestion (73.4%). A significant proportion of infants (70.2%) had dyspnea, while fever was present in one third of patients with RSV. In a retrospective study of children up to 5 years of age with RSV in Germany, the majority of hospitalized patients were infants and respiratory manifestations dominated the clinical picture, with dyspnea present in 79.6% of infants aged 0–6 months and 81.3% of infants aged 6 months to 1 year [24].

Our analysis shows clear differences in the clinical presentation of the two infections in infants. While RSV infection is more likely to present as a clinical respiratory syndrome with cough and/or dyspnea, SARS-CoV-2 infection may present as a polymorphic picture dominated by fever in most cases. Similar data were reported in a review by Fedorczak et al. for infants younger than 3 months only [25].

In our study, we observed that infants with SARS-CoV-2 infection presented to hospital earlier than those with RSV infection. This could be explained in particular by the more frequent presence of fever in those with COVID-19, as fever is known to be one of the clinical symptoms most feared by parents, especially in this age group. Hatmann et al. had similar results to ours, showing hospital presentation for children with RSV on average 3 days after the onset of symptoms [24].

In terms of laboratory investigations, the most common change described in both groups was an increase in monocytes. In particular, lymphocytosis was more common in infants with RSV and lymphopenia was more common in infants with SARS-CoV-2. Brüssow et al. [26] showed that the decreased lymphocyte count associated with SARS-CoV-2 infection could be explained by the inhibitory effect of activated cytokines. This also overlaps with our finding that elevated C-reactive protein values were more common in infants with COVID-19.

Our findings indicated a distinct difference in radiological presentations between infants suffering from COVID-19 and those with RSV. Specifically, infants afflicted with COVID-19 tended to exhibit normal imaging results more frequently. In contrast, those diagnosed with RSV were more prone to show signs of interstitial pneumonia in their radiological evaluations. This divergence in radiological findings could potentially be attributed to the greater likelihood of lower airway involvement in infants with RSV, as compared to those with COVID-19. This aspect of our study highlights the variances in how these two viral infections manifest in pediatric patients, particularly in terms of their impact on the respiratory system as observed through imaging studies.

COVID-19 infants had a shorter hospital stay than those with RSV, in whom we observed that the presence of acute respiratory failure increased the length of hospitalization by at least 3 days. Our results are consistent with data from other studies that have highlighted the need for longer hospital stays and more complex management among children with RSV compared to SARS-CoV-2 [25,27]. In our analysis focusing on the length of hospital stay in different age groups, we found that the median length of hospital stay did not vary significantly between groups. This observation suggests a relatively consistent pattern in the length of hospital stay across different age groups of infants. However, it is noteworthy that the shortest median length of hospital stay was specifically observed in the group of newborns infected with COVID-19. This aspect of our analysis provides valuable insights into how the duration of hospitalization for COVID-19 may differ in the earliest stages of life compared to older infants.

The outcome of all infants was positive and there were no deaths. Infants infected with RSV had a higher risk of developing respiratory failure, while those infected with COVID-19 had a higher risk of laryngitis. The results from other trials also suggest a favorable outcome for both infections [25,27], but the most common complication observed in the short term was the suspicion of bacterial superinfection [25].

In the current context of COVID-19 pandemic development, vigilant surveillance of RSV and SARS-CoV-2, along with influenza and other respiratory viruses, is extremely important [28,29]. These viruses pose significant public health challenges in terms of high numbers of patients seeking medical care, high rates of hospitalization and high numbers of deaths. In addition to its known impact on infants and young children, RSV is now known to be an important respiratory pathogen in older adults, particularly those with associated chronic diseases, and can lead to decompensation and progression with severe complications. At the same time, SARS-CoV-2 has demonstrated its capacity for rapid mutation, leading to various circulant variants with differing levels of transmissibility and severity. Persistent surveillance is crucial for the early detection of new variants, which is essential for timely public health responses, including updates to vaccines and treatment protocols.

Surveillance also plays a critical role in understanding the epidemiology of these viruses, such as transmission dynamics, infection rates, and population immunity. This information is vital for guiding public health policies, including vaccination strategies, social distancing measures, and resource allocation for healthcare facilities. Concurrent surveillance of RSV and SARS-CoV-2 is vital due to the potential for co-infections, which can exacerbate the severity of illness and complicate treatment strategies. Such surveillance helps in preparing healthcare systems for potential surges in cases, ensuring adequate medical supplies, and informing the public about preventive measures.

Although our study provides valuable insights, it has several limitations, mainly due to its retrospective design and the lack of extended follow-up of the infants included in our analysis. Another notable limitation of our study is the relatively small sample size of patients. A limited number of participants may reduce the statistical power of the study, making it more difficult to detect significant differences or draw robust conclusions. This small sample size may also limit the generalizability of our findings to the wider population. However, it is important to highlight a strength of our study, which is the careful matching of SARS-CoV-2 and RSV cases. By rigorously matching these cases, our study provides a clearer and more detailed comparison of the differences between the two infections in infants. This aspect of our methodology increases the reliability of our findings in understanding how these infections differ, particularly in their clinical presentation, severity, and short-term outcomes in this vulnerable age group.

## 5. Conclusions

The findings of this study suggest that infants with SARS-CoV-2 could be discriminated by clinical phenotyping from those with RSV infection, in the initial triage approach in an emergency department in order to have optimal hospital management, through the use of a tailored viral detection strategy, especially in a low-resource setting where monoclonal antibodies or maternal RSV vaccine prophylaxis are not available. Infants with SARS-CoV-2 infection may have polymorphic symptoms, mostly dominated by fever, whereas infants with RSV often have a cough and/or shortness of breath. Continued surveillance of both viruses in infants, and beyond, and the implementation of specific control measures are needed to mitigate their impact on this vulnerable pediatric group, according to dynamic, changing post-COVID-19 pandemic seasonal circulation.

## Figures and Tables

**Table 1:** Demographic characteristics of the groups analyzed.

Characteristic	SARS-CoV-2 Infection, *n* (%)	RSV Infection, *n* (%)	Total, *n* (%)
Sex			
Male	54 (57.4)	54 (57.4)	108 (57.4)
Female	40 (42.6)	40 (42.6)	80 (42.6)
Age group			
0–28 days	9 (9.6)	9 (9.6)	18 (9.6)
1–3 months	45 (47.9)	45 (47.9)	90 (47.9)
4–6 months	19 (20.2)	19 (20.2)	38 (20.2)
7–9 months	13 (13.8)	13 (13.8)	26 (13.8)
10–12 months	8 (8.5)	8 (8.5)	16 (8.5)
Median age (IQR), in months	3 (IQR: 2.0, 5.8)	3 (IQR: 1.0, 6.0)	3 (IQR: 1.0, 6.0)

RSV—respiratory syncytial virus.

**Table 2:** Comparison of the clinical characteristics between the groups.

Clinical Characteristic	SARS-CoV-2 Infection, N = 94, *n* (%)	RSV Infection, N = 94, *n* (%)	Statistical Analysis
Fever	77 (81.9)	29 (30.9)	*p* < 0.001, χ^2^ = 49.8, OR = 10.2, 95%CI: 5.12–20.11
Maximum temperature, °C, median (IQR)	38.8 (IQR: 38.5–39.3)	38.5 (IQR: 38.3–38.9)	*p* = 0.039, U = 621, z = −2.061, r = 0.150
Cough	64 (68.1)	94 (100.0)	*p* < 0.001, χ^2^ = 35.7, OR = 0.5, 95%CI: 0.25–0.82
Nasal congestion	45 (47.9)	69 (73.4)	*p* < 0.001, χ^2^ = 12.8, OR = 0.3, 95%CI: 0.2–0.6
Dyspnea	13 (13.8)	66 (70.2)	*p* < 0.001, χ^2^ = 61.3, OR = 0.07, 95%CI: 0.03–0.14
Diarrhea	29 (30.9)	15 (16.0)	*p* = 0.016, χ^2^ = 5.8, OR = 2.4, 95%CI: 1.16–4.75
Vomiting	12 (12.8)	14 (14.9)	*p* = 0.673, χ^2^ = 0.2, OR = 0.8, 95%CI: 0.37–1.91
Malaise	15 (16.0)	31 (33.0)	*p* = 0.007, χ^2^ = 7.3, OR = 0.4, 95%CI: 0.19–0.78

RSV—respiratory syncytial virus.

**Table 3:** Comparison of the laboratory parameters between the groups.

Laboratory Parameter	SARS-CoV-2 Infection, N = 94, *n* (%)	RSV Infection, N = 94, *n* (%)	Statistical Analysis
Increased white blood cell count	18 (19.1)	18 (19.1)	*p* = 1.000, χ^2^ = 0.0, OR = 1.0, 95%CI: 0.48–2.07
Decreased white blood cell count	10 (10.6)	7 (7.4)	*p* = 0.446, χ^2^ = 0.6, OR = 1.5, 95%CI: 0.53–4.07
Increased neutrophil count	5 (5.3)	1 (1.1)	*p* = 0.097, χ^2^ = 2.8, OR = 5.2, 95%CI: 0.60–45.61
Decreased neutrophil count	24 (25.5)	18 (19.1)	*p* = 0.293, χ^2^ = 1.1, OR = 1.5, 95%CI: 0.73–2.89
Increased monocyte count	78 (83.0)	76 (80.9)	*p* = 0.705, χ^2^ = 0.1, OR = 1.2, 95%CI: 0.55–2.43
Increased lymphocyte count	33 (35.1)	51 (54.3)	*p* = 0.008, χ^2^ = 6.9, OR = 0.5, 95%CI: 0.25–0.82
Decreased lymphocyte count	19 (20.2)	7 (7.4)	*p* = 0.011, χ^2^ = 6.4, OR = 3.1, 95%CI: 1.26–7.90
Increased C-reactive protein	31 (33.0)	15 (16.0)	*p* = 0.007, χ^2^ = 7.4, OR = 2.6, 95%CI: 1.29–5.22
Increased AST	12 (12.8)	48 (51.1)	*p* < 0.001, χ^2^ = 31.7, OR = 0.14, 95%CI: 0.07–0.29
Increased ALT	8 (8.5)	9 (9.6)	*p* = 0.799 χ^2^ = 0.1, OR = 0.8, 95%CI: 0.32–2.38
Increased urea	7 (7.4)	0 (0.0)	NA
Increased creatinine	1 (1.1)	0 (0.0)	NA
Chest X-ray			
Normal	11 (57.9)	2 (16.7)	*p* = 0.023, χ^2^ = 5.1, OR = 0.3, 95%CI: 0.05–0.8
Interstitial pneumonia	8 (47.1)	9 (52.9)	*p* = 0.806, χ^2^ = 0.1, OR = 0.9, 95%CI: 0.32–2.38
Lung consolidation	0 (0.0)	1 (8.3)	NA

AST—aspartate aminotransferase; ALT—alanine aminotransferase; NA—not applicable; RSV—respiratory syncytial virus.

## Data Availability

The datasets generated and analysed during the current study are available from the corresponding author upon reasonable request.

## Supplementary Materials

The following are available online at: https://www.mdpi.com/article/10.3390/v16010063/s1. Table S1. Patient characteristics by age group and type of infection.
