# Supplementary material for: Clinical Differences between SARS-CoV-2 and RSV Infections in Infants: Findings from a Case–Control Study
Source: Viruses. 2023 Dec 30;16(1):63. doi: 10.3390/v16010063 (PMC10819890; doi:10.3390/v16010063)
Supplement: Supplementary file 1 [file viruses-16-00063-s001.zip › viruses-2794588-supplementary.pdf]

Supplementary material. **Table S1.** Patient characteristics by age group and type of infection

	Age group	SARS-CoV-2 infection, n/N (%)	RSV infection, n/N (%)	p-value	OR	95%CI
Fever	Newborn	5/9 (55.6)	1/9 (11.1)	0.046	10.0	0.85-117.01
Fever	1-3 months	**38/45 (84.4)**	7/45 (15.6)	**<0.001**	29.5	9.42-92.20
Fever	4-6 months	**15/19 (78.9)**	9/19 (47.4)	**0.044**	4.2	1.003-17.31
Fever	7-9 months	**12/13 (92.3)**	7/13 (53.8)	**0.027**	10.3	1.01-103.95
Fever	10-12 months	7/8 (87.5)	5/8 (62.5)	0.248	4.2	0.33-53.12
Cough	Newborn	3/9 (33.3)	**9/9 (100)**	**0.003**	0.3	0.13-0.84
Cough	1-3 months	26/45 (57.8)	**45/45 (100)**	**<0.001**	0.6	0.45-0.76
Cough	4-6 months	16/19 (84.2)	19/19 (100)	0.071	0.8	0.69-1.02
Cough	7-9 months	12/13 (92.3)	13/13 (100)	0.308	0.9	0.79-1.08
Cough	10-12 months	7/8 (87.5)	8/8 (100)	0.302	0.9	0.67-1.14
Nasal congestion	Newborn	2/9 (22.2)	**8/9 (88.9)**	**0.004**	0.4	0.003-0.48
Nasal congestion	1-3 months	19/45 (42.2)	**29/45 (64.4)**	**0.035**	0.4	0.17-0.94
Nasal congestion	4-6 months	11/19 (57.9)	**18/19 (94.7)**	**0.008**	0.1	0.008-0.70
Nasal congestion	7-9 months	9/13 (69.2)	7/13 (53.8)	0.420	1.9	0.39-9.60
Nasal congestion	10-12 months	4/8 (50.0)	7/8 (87.5)	0.106	0.1	0.01-1.76
Dispnee	Newborn	0/9 (0.0)	**7/9 (77.8)**	**0.001**	0.2	0.07-0.75
Dispnee	1-3 months	6/45 (13.3)	**33/45 (73.3)**	**<0.001**	0.6	0.02-0.17
Dispnee	4-6 months	3/19 (15.8)	**11/19 (57.9)**	**0.007**	0.1	0.03-0.63
Dispnee	7-9 months	2/13 (15.4)	**9/13 (69.2)**	**0.005**	0.1	0.01-0.55
Dispnee	10-12 months	2/8 (25.0)	6/8 (75.0)	0.046	0.1	0.01-1.07
Diaree	Newborn	2/9 (22.2)	0/9 (0.0)	0.134	1.3	0.90-1.82
Diaree	1-3 months	**19/45 (42.2)**	7/45 (15.6)	**0.005**	4.0	1.46-10.78
Diaree	4-6 months	3/19 (15.8)	5/19 (26.3)	0.426	0.5	0.11-2.60
Diaree	7-9 months	3/13 (23.1)	3/13 (23.1)	1.000	1.0	0.16-6.2
Diaree	10-12 months	2/8 (25.0)	0/8 (0.0)	0.131	1.3	0.89-1.99
Vărsături	Newborn	1/9 (11.1)	0/9 (0.0)	0.303	1.1	0.89-1.41
Vărsături	1-3 months	4/45 (8.9)	8/45 (17.8)	0.215	0.5	0.13-1.62
Vărsături	4-6 months	3/19 (15.8)	3/19 (15.8)	1.000	1.0	0.18-5.72
Vărsături	7-9 months	3/13 (23.1)	2/13 (15.4)	0.619	1.7	0.23-11.99
Vărsături	10-12 months	1/8 (12.5)	1/8 (12.5)	1.000	1.0	0.05-19.36
Malaise	Newborn	1/9 (11.1)	2/9 (22.2)	0.527	0.4	0.03-5.29
Malaise	1-3 months	6/45 (13.3)	**18/45 (40.0)**	**0.004**	0.2	0.08-0.66
Malaise	4-6 months	2/19 (10.5)	3/19 (15.8)	0.631	0.6	0.09-4.26
Malaise	7-9 months	3/13 (23.1)	3/13 (23.1)	1.000	1.0	0.16-6.20
Malaise	10-12 months	3/8 (37.5)	5/8 (62.5)	0.317	0.4	0.05-2.73
Epidemiolo-gical context	Newborn	4/9 (44.4)	5/9 (55.6)	0.637	0.6	0.10-4.10
Epidemiolo-gical context	1-3 months	**27/45 (60.0)**	16/45 (35.6)	**0.020**	2.7	1.16-6.38
Epidemiolo-gical context	4-6 months	**9/19 (47.4)**	3/19 (15.8)	**0.036**	4.8	1.04-22.10
Epidemiolo-gical context	7-9 months	4/13 (30.8)	8/13 (61.5)	0.116	0.3	0.06-1.41
Epidemiolo-gical context	10-12 months	4/8 (50.0)	2/8 (25.0)	0.302	3.0	0.36-24.92

Increased white blood cells count	Newborn	0/9 (0.0)	3/9 (33.3)	0.058	0.7	0.42-1.05
	1-3 months	4/45 (8.9)	6/45 (13.3)	0.502	0.6	0.17-2.42
	4-6 months	4/19 (21.1)	6/19 (31.6)	0.461	0.6	0.13-2.51
	7-9 months	4/13 (30.8)	2/13 (15.4)	0.352	2.4	0.36-16.55
	10-12 months	**6/8 (75.0)**	1/8 (12.5)	**0.012**	21.0	1.50-293.25
Decreased white blood cells count	Newborn	1/9 (11.1)	0/9 (0.0)	NA	NA	NA
	1-3 months	6/45 (13.3)	2/45 (4.4)	0.138	3.3	0.63-17.36
	4-6 months	3/19 (15.8)	1/19 (5.3)	0.290	3.4	0.32-35.79
	7-9 months	0/13 (0.0)	2/13 (15.4)	NA	NA	NA
	10-12 months	0/8 (0.0)	2/8 (25.0)	NA	NA	NA
Increased neutrophil count	Newborn	0/9 (0.0)	0/9 (0.0)	NA	NA	NA
	1-3 months	0/45 (0.0)	0/45 (0.0)	NA	NA	NA
	4-6 months	1/19 (5.3)	0/19 (0.0)	NA	NA	NA
	7-9 months	2/13 (15.4)	1/13 (7.7)	0.539	2.2	0.17-27.56
	10-12 months	2/8 (25.0)	0/8 (0.0)	NA	NA	NA
Decreased neutrophil count	Newborn	4/9 (44.4)	1/9 (11.1)	0.114	6.4	0.54-74-89
	1-3 months	17/45 (37.8)	10/45 (22.2)	0.107	2.1	0.84-5.36
	4-6 months	2/19 (10.5)	3/19 (15.8)	0.631	0.6	0.09-4.26
	7-9 months	1/13 (7.7)	2/13 (15.4)	0.539	0.5	0.04-5.79
	10-12 months	0/8 (0.0)	2/8 (25.0)	NA	NA	NA
Increased monocyte count	Newborn	8/9 (88.9)	8/9 (88.9)	1.000	1.0	0.05-18.92
	1-3 months	34/45 (75.6)	39/45 (86.7)	0.178	0.5	0.16-1.42
	4-6 months	16/19 (84.2)	15/19 (78.9)	0.676	1.4	0.27-7.44
	7-9 months	12/13 (92.3)	11/13 (84.6)	0.539	2.2	0.17-27.56
	10-12 months	**8/8 (100)**	3/8 (37.5)	**0.007**	2.7	1.09-6.53
Increased lymphocyte count	Newborn	3/9 (33.3)	6/9 (66.7)	0.157	0.3	0.04-1.78
	1-3 months	14/45 (31.1)	**25/45 (55.6)**	**0.019**	0.4	0.15-0.86
	4-6 months	7/19 (36.8)	**15/19 (78.9)**	**0.009**	0.2	0.04-0.66
	7-9 months	4/13 (30.8)	4/13 (30.8)	1.000	1.0	0.19-5.29
	10-12 months	5/8 (62.5)	1/8 (12.5)	0.039	11.7	0.92-147.56
Decreased lymphocyte count	Newborn	1/9 (11.1)	1/9 (11.1)	1.000	1.0	0.05-18.92
	1-3 months	**12/45 (26.7)**	1/45 (2.2)	**0.001**	16.0	1.98-129.2
	4-6 months	6/19 (31.6)	1/19 (5.3)	0.036	8.3	0.89-77.57
	7-9 months	0/13 (0.0)	1/13 (7.7)	NA	NA	NA
	10-12 months	0/8 (0.0)	3/8 (37.5)	NA	NA	NA
Increased C-reactive protein	Newborn	0/9 (0.0)	1/9 (11.1)	NA	NA	NA
	1-3 months	8/45 (17.8)	6/45 (13.3)	0.561	1.4	0.45-4.44
	4-6 months	**9/19 (47.4)**	1/19 (5.3)	**0.003**	16.2	1.79-147.07
	7-9 months	8/13 (61.5)	5/13 (38.5)	0.239	2.6	0.53-12.43
	10-12 months	6/8 (75.0)	2/8 (25.0)	0.046	9.0	0.94-86.52
Increased AST	Newborn	2/9 (22.2)	3/9 (33.3)	0.599	0.6	0.07-4.64
	1-3 months	5/45 (11.1)	**18/45 (40.0)**	**0.002**	0.2	0.06-0.57
	4-6 months	3/19 (15.8)	**13/19 (68.4)**	**0.001**	0.1	0.02-0.42
	7-9 months	1/13 (7.7)	**8/13 (61.5)**	**0.004**	0.05	0.005-0.53
	10-12 months	1/8 (12.5)	**6/8 (75.0)**	**0.012**	0.05	0.003-0.67

Increased ALT	Newborn	1/9 (11.1)	0/9 (0.0)	NA	NA	NA
	1-3 months	4/45 (8.9)	5/45 (11.1)	0.725	0.78	0.20-3.12
	4-6 months	2/19 (10.5)	3/19 (15.8)	0.631	0.63	0.09-4.26
	7-9 months	0/13 (0.0)	1/13 (7.7)	NA	NA	NA
	10-12 months	1/8 (12.5)	0/8 (0.0)	NA	NA	NA
Increased urea	Newborn	1/9 (11.1)	0/9 (0.0)	NA	NA	NA
	1-3 months	2/45 (4.4)	0/45 (0.0)	NA	NA	NA
	4-6 months	0/19 (0.0)	0/19 (0.0)	NA	NA	NA
	7-9 months	3/13 (23.1)	0/13 (0.0)	NA	NA	NA
	10-12 months	1/8 (12.5)	0/8 (0.0)	NA	NA	NA
Increased creatinine	Newborn	0/9 (0.0)	0/9 (0.0)	NA	NA	NA
	1-3 months	0/45 (0.0)	0/45 (0.0)	NA	NA	NA
	4-6 months	0/19 (0.0)	0/19 (0.0)	NA	NA	NA
	7-9 months	0/13 (0.0)	0/13 (0.0)	NA	NA	NA
	10-12 months	1/8 (12.5)	0/8 (0.0)	NA	NA	NA
Acute respiratory failure	Newborn	0/9 (0.0)	**4/9 (44.4)**	**0.023**	0.56	0.31-0.99
	1-3 months	1/45 (2.2)	**20/45 (44.4)**	**<0.001**	0.03	0.004-0.23
	4-6 months	0/19 (0.0)	3/19 (15.8)	0.071	0.84	0.69-1.02
	7-9 months	1/13 (7.7)	5/13 (38.5)	0.063	0.13	0.01-1.37
	10-12 months	0/8 (0.0)	3/8 (37.5)	0.055	0.63	0.37-1.07
Laryngitis	Newborn	0/9 (0.0)	0/9 (0.0)	NA	NA	NA
	1-3 months	0/45 (0.0)	1/45 (2.2)	NA	NA	NA
	4-6 months	**8/19 (42.1)**	2/19 (10.5)	**0.027**	6.18	1.10-34.70
	7-9 months	**5/13 (38.5)**	0/13 (0.0)	**0.013**	1.63	1.06-2.50
	10-12 months	2/8 (25.0)	0/8 (0.0)	NA	NA	NA
Days of hospitalization	Newborn	3 days (IQR: 0.5, 4.5)	5 days (IQR: 3.0, 6.0)	0.136	NA	NA
	1-3 months	5 days (IQR: 3.0, 7.0)	6 days (IQR: 4.0, 8.0)	0.067	NA	NA
	4-6 months	5 days (IQR: 3.0, 6.0)	4 days (IQR: 2.0, 5.0)	0.116	NA	NA
	7-9 months	4 days (IQR: 2.5, 6.5)	5 days (IQR: 4.0, 7.0)	0.336	NA	NA
	10-12 months	4.5 days (IQR: 2.5, 7.0)	5.5 days (IQR: 3.5, 8.5)	0.442	NA	NA

AST - aspartate aminotransferase, ALT - alanine aminotransferase; NA - Not applicable;
RSV - respiratory syncytial virus.
In bold black p-values with statistical significance. In bold blue the group with statistical significance
